# Temperature-Driven Textural Evolution, Quality Changes, and Flavor Development in Luncheon Meat

**DOI:** 10.3390/foods15173131

**Published:** 2026-09-03

**Authors:** Minnan Liu, Wanli Zhang, Jiaqi Sun, Yingjian Hou, Shuanshuan Xue, Zhenxia Cao, Bing Yang, Jing Yan, Heng Wang, Lishui Chen

**Affiliations:** Food Laboratory of Zhongyuan, Luohe 462300, China

**Keywords:** luncheon meat, flavor, quality

## Abstract

Different heat treatment temperatures (85–115 °C) were evaluated for their effects on the quality and flavor of luncheon meat. As the heating temperature increased, the weight loss rate increased, and its color became darker. Low-field nuclear magnetic resonance (LF-NMR) revealed that immobilized water decreased progressively while bound and free water both increased. During heat treatment, alterations in the spatial structure of proteins reduced the hardness and chewiness of luncheon meat, accompanied by significant variations in amino acid contents. The MDA content increased from 0.34 to 0.69 mg/kg with increasing temperature, confirming enhanced lipid oxidation. Through gas chromatography–ion mobility spectrometry (GC-IMS) analysis, a total of 38 volatile organic compounds (VOCs) were identified, primarily aldehydes, ketones, and esters; the total aldehydes content increased by 57.5% from 85 °C to 115 °C. Combining odor activity values (OAV > 1) with orthogonal partial least squares-discriminant analysis (OPLS-DA, VIP > 1), 9 core flavor compounds were identified. Heating at 95 °C balanced aldehyde–ester flavor development with minimal quality loss. This study provides key theoretical foundations for optimizing heat treatment parameters in the industrial production of luncheon meat.

## 1. Introduction

As a classic emulsified meat product, luncheon meat has a broad consumer base worldwide due to its ease of storage, convenience, unique flavor and texture [1,2]. Luncheon meat is typically made by mixing pork with starch, protein, seasonings, and compound additives, then packaging the mixture in cans or casings and subjecting it to heat treatment [1]. During the production of luncheon meat, heat treatment inactivates or inhibits microorganisms, thereby ensuring consumer health and safety. Moreover, the heat treatment step is a critical determinant of the final product’s quality, as it governs protein gelation, water retention, and flavor formation—all of which directly influence consumer acceptance [3].

From a physicochemical perspective, heat treatment triggers a series of reactions, including fatty acid degradation, protein denaturation, Strecker degradation, and thiamine thermal degradation [4]. Among these, protein denaturation plays a central role in texture formation: myofibrillar proteins, which account for 50–55% of total meat proteins and possess heat-induced gelation properties, undergo conformational unfolding, aggregation, and oxidative cross-linking during heating, thereby modifying the architecture of the gel network [5,6,7]. Moderate heating results in the formation of a tight network structure of myofibrillar proteins, whereas excessive heating may lead to protein degradation and excessive aggregation, thereby causing deterioration in product texture [8]. Heating generally lowers the water-holding capacity (WHC) of meat, and both the end-point temperature and the heating rate determine the extent of cooking loss [9]. Palka and Daun [10] reported that cooking losses of bovine semitendinosus increased continuously as the end-point temperature was raised from 50 to 121 °C, whereas texture profile analysis (TPA) hardness and chewiness first increased and then decreased. Chiavaro et al. [11] observed that raising the cooking temperature of pork Longissimus dorsi from 100 to 140 °C progressively increased cooking loss, decreased WHC, and produced paler and less tender products. Pang et al. [12] found that at higher cooking temperatures (>57 °C), the loss of moisture from myofibrils increased, resulting in a higher loss rate of chicken during cooking. Zhang et al. [13] found that as heat treatment temperature increased, the cooking loss and yellowness of duck meat increased. With respect to texture, prolonged heating decreased the hardness, elasticity, and chewiness of duck meat [13]. Zhu et al. [8] similarly observed that when pork sausages were heated at 90–120 °C for 20 min, their hardness initially increased and then decreased. Furthermore, rising heat treatment temperatures led to reduced tenderness and increased cooking loss in beef [14]. Becker et al. [15] found that pork heated at 53 °C or 58 °C for over 20 h was more tender and had a higher moisture content than pork heated using traditional methods. Low temperature alleviates heat stress in myofibrillar conformation and reduces protein aggregation [16]. With respect to sensory quality, duck meat cooked under moderate conditions (70 °C for 6 or 12 h) exhibited better sensory characteristics [13]. Kim et al. [17] found that higher F0 significantly increased shear force and lipid oxidation, while reducing polyunsaturated fatty acid content. From an industrial perspective, choosing the thermal treatment temperature essentially involves a trade-off between microbial safety and edible quality. The core objective of commercial thermal treatment is to achieve the target lethality rate while maximizing the protection of sensory and nutritional properties [18].

In terms of flavor formation, heat treatment temperature directly determines the characteristic aroma profile of meat products through the regulation of Maillard reaction, lipid oxidation and thermal degradation of thiamine, and its industrial control has always been a research hotspot in the field of meat processing [19]. Xu et al. [20] systematically reviewed the mechanisms and industrial applications of flavor formation during meat product thermal processing, stating that temperature is one of the key parameters affecting the types and intensities of volatile compounds generated, and moderate heating promotes the formation of characteristic aroma compounds in meat. In this context, the applied temperature largely determines the direction and extent of flavor development. Pan et al. [21] reported that appropriate thermal treatment (80 °C and 95 °C) of spiced beef enhanced the contents of compounds associated with roasted meat flavor (e.g., 3-methylbutanal, 1-octen-3-ol) and caramel-like flavor compounds (e.g., hydroxyacetone), and produced 2-methylthiophene and methyl disulfide at 110 °C. Similar findings have also been observed in cooked pork and duck meat. Zhu et al. [8] reported that sausages cooked at 120 °C exhibited significantly stronger fatty and roasted flavors, whereas flower and fruity flavors decreased, compared with sausages cooked at 90 °C. Xie et al. [22] found that the overall flavor of boiled salted duck decreased after high-temperature sterilization (121 °C for 20 min), due to the accumulation of aldehydes and ketones produced by lipid oxidation caused by excessive heating. Furthermore, in addition to affecting product quality and flavor, excessive heating may also lead to the formation of harmful substances, such as heterocyclic amines [23]. In recent years, the analysis of VOCs in foods using various technical methods has become a research hotspot. Gas chromatography–olfactometry–mass spectrometry, GC-IMS, and E-nose are the main techniques employed for identifying flavor compounds in meat products [24,25,26].

The commonly used heat treatment temperatures in food processing include pasteurization (65–90 °C), commercial sterilization (110–121 °C), and ultra-high temperature sterilization (140–160 °C) [21]. At present, pasteurization and commercial sterilization are widely adopted for luncheon meat products, which are categorized into low-temperature luncheon meat and ambient-temperature luncheon meat according to the applied sterilization temperature. Commercially, ambient-temperature sliced luncheon meat is produced through a two-step thermal process: (1) initial cooking to form a stable emulsion gel network and ensure product integrity, and (2) post-packaging thermal processing above 100 °C to achieve commercial sterility and shelf stability. This study focuses on the second thermal processing step, examining how temperatures ranging from 85 to 115 °C affect product quality and flavor characteristics. Although numerous studies have examined the effects of heat treatment on various meat products, systematic research specifically addressing luncheon meat remains limited. Luncheon meat differs from other emulsified meat products in its unique formulation, thermal processing, and packaging format. These differences may result in distinct quality and flavor changes in response to thermal processing. Therefore, this study investigated the effects of different thermal processing temperatures (85–115 °C) on the quality and flavor of luncheon meat. To evaluate its quality characteristics, color, texture properties, water distribution, and amino acid composition were determined. In addition, the flavor characteristics of the luncheon meat were analyzed using GC-IMS and E-nose.

## 2. Materials and Methods

### 2.1. Materials

Pork foreleg meat (74.3% moisture, 21.4% protein, and 4.9% fat) and pork fat (1.93% protein and 88.9% fat) were purchased from a local meat supplier (Luohe, China). Pork foreleg refers to the boneless muscle from the foreleg section cut from between the fifth and sixth ribs of the pig carcass. The strains (*Staphylococcus carnosus*, *Mammaliicoccus vitulinus*), the seasoning and collagen protein seasoning powder (including collagen, maltodextrin, soup powder, and salt) were purchased from Suzhou Wen Da Food Ingredient Co., Ltd. (Kunshan, China). The seasoning is a commercial blend whose main ingredients include trehalose, sodium D-isoascorbate, beet juice powder, salt, and maltodextrin as a carrier. The phosphate (including sodium pyrophosphate, sodium tripolyphosphate and sodium hexametaphosphate) was purchased from Hubei Xingfa Chemical Group Co., Ltd. (Yichang, China). Salt, corn starch, white sugar, white pepper, transglutaminase, nisin, and food-grade plastic casings were purchased from commercial food-ingredient suppliers. All ingredients were food grade.

### 2.2. Production of Luncheon Meat

**Figure 1:**
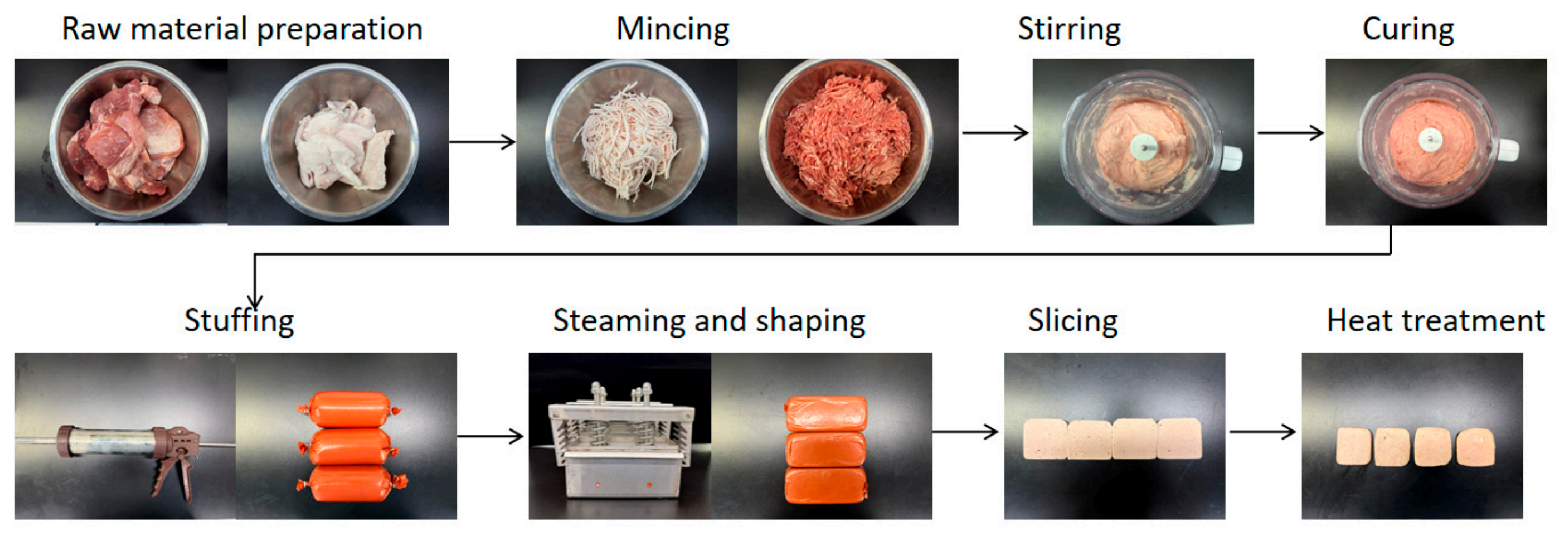
The production process of luncheon meat.

The basic formulation for luncheon meat preparation is listed in Table 1. First, the pork foreleg meat and pork fat were ground using a meat grinder (with a 4 mm plate). The minced foreleg meat and fat were chopped in a 2 L plastic processing bowl (Model K600) matched with a Braun Tribute Collection food processor (Model FP3010, Type 3205, De’Longhi-Braun-Household GmbH, Neu-Isenburg, Germany). The mixture was chopped at speed setting 2 for 3 min, followed by phosphate addition and another 10 min of chopping at the same speed. Salt and 80 g ice water (to regulate batter temperature) were then incorporated and mixed at speed 5 for 8 min, before adding corn starch and bacterial suspension (bacterial strain and composite seasonings dissolved in 20 g ice water) and homogenizing at speed 1 for 3 min. The batter was marinated at 4 °C for 18 h, then blended with residual ingredients and chopped at speed 3 for 3 min. The minced meat was then injected into plastic sausage casings using a sausage injector and placed in a food-grade meat mold made of 304 stainless steel (140 mm × 60 mm × 60 mm). Each filled segment was approximately 125 mm in length, yielding a weight of approximately 430 g per segment. Next, the samples were heat-treated in an 85 °C water bath for 60 min, then immersed in cold water to rapidly cool them to room temperature before being sliced into 1 cm thick pieces. The slices were then vacuum-packed and heat-treated at different temperatures (85, 95, 105, and 115 °C) for 20 min, followed by cooling in cold water to room temperature (25 ± 2 °C). The 85 °C and 95 °C treatments were conducted in a constant-temperature water bath, while the 105 °C and 115 °C treatments were performed in a high-pressure steam sterilizer to ensure that the temperature exceeded the boiling point of water. For each treatment temperature, three independent samples of luncheon meat were prepared as biological replicates. The production process of luncheon meat is shown in Figure 1.

### 2.3. Determination of Weight Loss Rate

The weight loss rate (%) was calculated as the ratio of the weight change before and after heat treatment to the weight before heat treatment [27].

### 2.4. Color Determination

The color of each sample was measured at three randomly selected surface locations using a portable colorimeter (CR-400, Konica Minolta, Tokyo, Japan) calibrated with a white standard calibration plate [28]. The CIELAB color parameters L*, a*, and b* were recorded under illuminant D65 with a 10° standard observer angle and an 8 mm measurement aperture.

### 2.5. Texture Profile Analysis (TPA)

TPA was performed using a method slightly modified from that reported by Zhao et al. [29]. Samples were cut into 1 × 1 × 1 cm cubes and compressed twice using a P36 probe on a texture analyzer (CTX, AMETEK Brookfield, Middleborough, MA, USA) at 40% deformation. The pre-test speed was 2 mm/s; both the test and post-test speeds were 1 mm/s; the trigger force was 3 g. All measurements were performed in 10 replicates. The following texture parameters were determined: hardness, cohesiveness, springiness, gumminess, and chewiness.

### 2.6. Water Distribution by LF-NMR

The sample (2 g) was wrapped with sterile tape to prevent water loss and placed in a 25 mm nuclear magnetic tube [30]. The transverse relaxation time was determined using the Carr–Purcell–Meiboom–Gill (CPMG) pulse sequence. The sampling interval was 2000 ms, the echo count was 10,000 times, the echo time was 0.1 ms, and the sampling frequency was 4 times. The multi-index inversion fitting was carried out using the dedicated analysis software for the instrument, with the number of iterations set at 100,000 times.

### 2.7. Determination of Amino Acid Content

Total amino acids (TAA) in the samples were determined in accordance with GB 5009.124-2016 [31]. In brief, homogenized samples (0.1 g) were transferred into heat-resistant glass tubes with screw caps, followed by the addition of 15 mL of 6 mol/L HCl and a few drops of phenol solution. The tubes were purged with nitrogen gas, sealed, and placed in an oven at 110 °C for 22 h. After cooling to room temperature, the hydrolysates were filtered through qualitative filter paper. A 1 mL portion of the filtrate was concentrated to dryness using a rotary evaporator, and the dried residue was redissolved in 1 mL of pH 2.2 sodium citrate buffer solution for subsequent instrumental analysis. A fully automatic amino acid analyzer (L-8900, Hitachi High-Tech, Tokyo, Japan) was employed for the separation and quantitative detection of amino acid components. Serially diluted mixed amino acid standard solutions were analyzed to establish standard curves for external standard quantitative calculation. The instrumental detection parameters were set as follows: separation column temperature of 57 °C, detection wavelength of 570 nm and 440 nm, buffer flow rate of 0.35 mL/min, and reaction unit temperature of 135 °C. All sample determinations were performed in triplicate to ensure experimental accuracy and reproducibility.

### 2.8. E-Nose Analysis

The minced samples (5.0 g) were sealed in a headspace sample bottle and incubated at 60 °C for 30 min to enrich volatile components. Then, the sample was tested using an E-nose (PEN3, AIRSENSE Analytics GmbH, Schwerin, Germany). Before each measurement, the sensor was pre-cleaned for 120 s with clean dry air, and then measurements were conducted for 80 s at a carrier gas flow rate of 0.4 L/min. Each sample was analyzed in triplicate. The E-nose was equipped with 10 gas sensors, which exhibited specific responses to different odors. The core response information of each sensor is presented in Table 2.

### 2.9. Determination of Malondialdehyde (MDA) Content

According to GB 5009.181-2016 [32], the MDA content was determined. 5.0 g of homogenized sample was accurately weighed, and 50 mL of 7.5% trichloroacetic acid (containing 0.1% disodium ethylenediaminetetraacetate, EDTA) was added. The mixture was shaken at 50 °C for 30 min, and then filtered. 5 mL of the filtrate was added to 5 mL of 0.02 mol/L thiobarbituric acid (TBA) solution, and the mixture was heated in a water bath at 90 °C for 30 min. After cooling, the absorbance was measured at 532 nm. A standard curve for MDA was prepared (0.01–0.25 μg/mL) for quantitative analysis. Each group was subjected to three parallel determinations.

### 2.10. Determination of VOCs

The VOCs in the samples were determined using GC-IMS (FlavourSpec^®^, G. A. S., Dortmund, Germany). A 3.0 g sample was weighed and 30 µL of 100 ppm 2-octanol was added. The sample injection procedure is detailed in the Supplementary Materials. The results were searched and matched using the VOCal 1 software, the IMS migration time database, and the NIST 2020 mass spectrometry database. The relevant spectra were plotted using the built-in plugin in this software. The n-ketones (C4–C9) were used as the external standard solution, and the calibration curve was determined under the same chromatographic conditions for the qualitative analysis of compounds. 2-Octanol was used as the internal standard for the semi-quantitative analysis of volatile compounds. The concentrations of each target compound were calculated based on the ratio of its peak volume to that of the internal standard peak, multiplied by the known concentration of 2-octanol. Three replicates were measured for each treatment group.

### 2.11. Calculation of Odor Activity Value (OAV)

The OAV of volatile flavor substances is calculated as the ratio of their actual concentration to their flavor threshold in water [33].

### 2.12. Statistical Analysis

Data were analyzed with SPSS 23.0; graphs were generated in Origin 2018. OPLS-DA was performed in SIMCA 14.1. The model was validated by a 200-time permutation test. Results are expressed as mean ± standard deviation (n = 3 independent experimental replicates per treatment, each analyzed in triplicate). Group differences were evaluated by one-way ANOVA followed by Duncan’s multiple-range test (*p* < 0.05).

## 3. Results and Discussion

### 3.1. Weight Loss Rate

The weight loss rate is one of the basic indicators for assessing the quality of luncheon meat, which can accurately reflect the ability to retain water and fat during thermal processing. The results in Table 3 showed that weight loss increased with increasing thermal treatment temperature, ranging from 2.63% to 3.26%. During thermal processing, meat undergoes substantial water loss, accompanied by the exudation of soluble proteins, lipids and non-protein soluble substances [14]. Wereńska et al. [34] reported that cooking loss of goose meat increased from 23.65% to 42.18% with increasing temperature. Moreover, a similar trend was observed in duck meat and beef [13,14]. Notably, the weight loss values obtained in the present study (2.63–3.26%) were far lower than the cooking losses typically reported for whole muscles. This can be attributed to the high-density protein–fat–starch matrix in the emulsified luncheon meat system: the heat-set gel network physically entraps water and fat within the product and largely suppresses the exudation otherwise induced by muscle-fiber shrinkage [35]. Previous studies have demonstrated that prolonged thermal treatment induces protein denaturation and cross-linking in meat products, leading to structural changes that may affect their overall texture and water-binding capacity [34]. From an industrial perspective, this parameter is of direct economic significance because weight loss determines the product yield—a lower weight loss increases the output of finished product per unit of raw material and thereby improves the economic benefit of luncheon meat production.

### 3.2. Color

Color measurement (a*, b*, and L*) is an indicator for quantifying color variations in foods. These instrumental color parameters have been demonstrated to correlate significantly with human visual evaluation and consumer acceptance of meat products. Holman et al. [36] discovered that consumers have a greater preference for red or bright red. Altmann et al. [37] pointed out that among all the CIELAB color dimensions, the change in the b* is the most obvious for consumers, followed by the a*, and finally the L*, highlighting the direct sensory correlation of b* changes. The effect of temperature on the color parameters of luncheon meat is shown in Table 3. The L* value remained between 58.05 and 59.40, while the values of a* and b* showed an overall upward trend. This change may be related to reactions occurring during heating, such as protein oxidation and denaturation, the Maillard reaction, and lipid oxidation. High-temperature heat treatment leads to the unfolding of globin peptide chains, and promotes the dissociation and oxidation of heme groups to form ferriheme, thereby significantly affecting the final color of meat products [34]. Therefore, the color changes caused by different heat treatment temperatures in luncheon meat not only reflect potential chemical reactions but also directly affect consumers’ visual acceptance and purchasing decisions.

### 3.3. TPA

Changes in the texture properties of luncheon meat during heating are presented in Table 4. With increasing temperature, the hardness, chewiness, and gumminess of luncheon meat decreased. A similar trend was reported by Mohamed et al. [38], who found that the shear force and hardness of sausages decreased as the cooking temperature increased. The marked decrease in hardness and chewiness may negatively affect consumer acceptability. During moderate heating, myofibrillar proteins and connective tissue proteins denature and associate through disulfide bond cross-linking, forming a dense gel network that tightly envelops fat droplets [38]. However, once this network has been established, exposure to higher temperatures induces protein degradation and over-aggregation, which disrupts and weakens the network structure and promotes the exudation of moisture and fat, thereby reducing the hardness of the product [8]. Nevertheless, as the temperature increased, the springiness of luncheon meat first increased and then decreased. Zhu et al. [8] found that the springiness of emulsified sausages first increased and then decreased with rising temperature. It has been reported that heat-induced denaturation of myofibrillar proteins and unfolding of polypeptide chains may expose hydrophobic binding sites buried within the protein interior, thereby increasing surface hydrophobicity [39]. In addition, heating promotes the oxidation of thiol groups in proteins to form disulfide bonds, facilitating inter- or intramolecular cross-linking, which results in a more compact arrangement of myofibrils [40]. Together, these changes form a gel network with higher elasticity, but excessive heating can disrupt this structure. In summary, protein denaturation, aggregation, and degradation are considered the primary causes of texture changes in meat products during heat treatment [41]. The hardness and springiness determine the sliceability, mouthfeel and overall eating quality of luncheon meat, and excessive softening impairs slicing performance, packaging appearance and consumer acceptance, leading to product downgrading and economic loss. Moreover, because even a 10 °C increase in temperature can significantly alter the texture of the final product, precise control of the sterilization temperature is essential to ensure consistency of texture between batches.

### 3.4. Water Distribution

LF-NMR is commonly used to evaluate changes in the distribution of water in meat products. There are generally three types of water present in muscle: (a) bound water (T_21_, 0.01–10 ms), which is tightly bound to macromolecules; (b) immobilized water (T_22_, 10–100 ms), mainly trapped within the myofibrillar and filament network; and (c) free water (T_23_, 100–1000 ms), located primarily in the extramyofibrillar spaces [30]. The relative proportion of each water fraction (A_21_, A_22_, and A_23_) was calculated as the ratio of the corresponding peak area to the total peak area of all three T2 components. As shown in Table 5, with increasing temperature, the content of bound water increased, indicating that high temperature promoted the binding between proteins and water molecules. As the most abundant water fraction, immobilized water showed a decreasing trend with rising temperature, whereas free water exhibited the opposite trend. This suggested that changes in the protein gel network induced the transformation of part of the immobilized water into other water states. The water-holding capacity of meat is significantly correlated to the mobility of water within the dense matrix of myofibrils [42]. Han et al. [43] discovered that with increasing temperature, immobilized water was converted to free water, and the proportion of water bound by proteins decreased.

### 3.5. Amino Acid Content

As shown in Table 6, the total amino acid (TAA) content of luncheon meat exhibited an initial increase followed by a decrease with increasing temperature, peaking at 95 °C (14.71 g/100 g) before declining to 14.37 and 14.31 g/100 g at 105 °C and 115 °C, respectively. The total essential amino acid (TEAA) content followed a similar trend, increasing from 5.17 g/100 g at 85 °C to 5.61 g/100 g at 95 °C, and subsequently decreasing to 5.43 g/100 g at both 105 °C and 115 °C. Previous studies have demonstrated that the decrease in total essential amino acid (TEAA) content indicates a decline in nutritional value [8].

### 3.6. E-Nose

**Figure 2:**
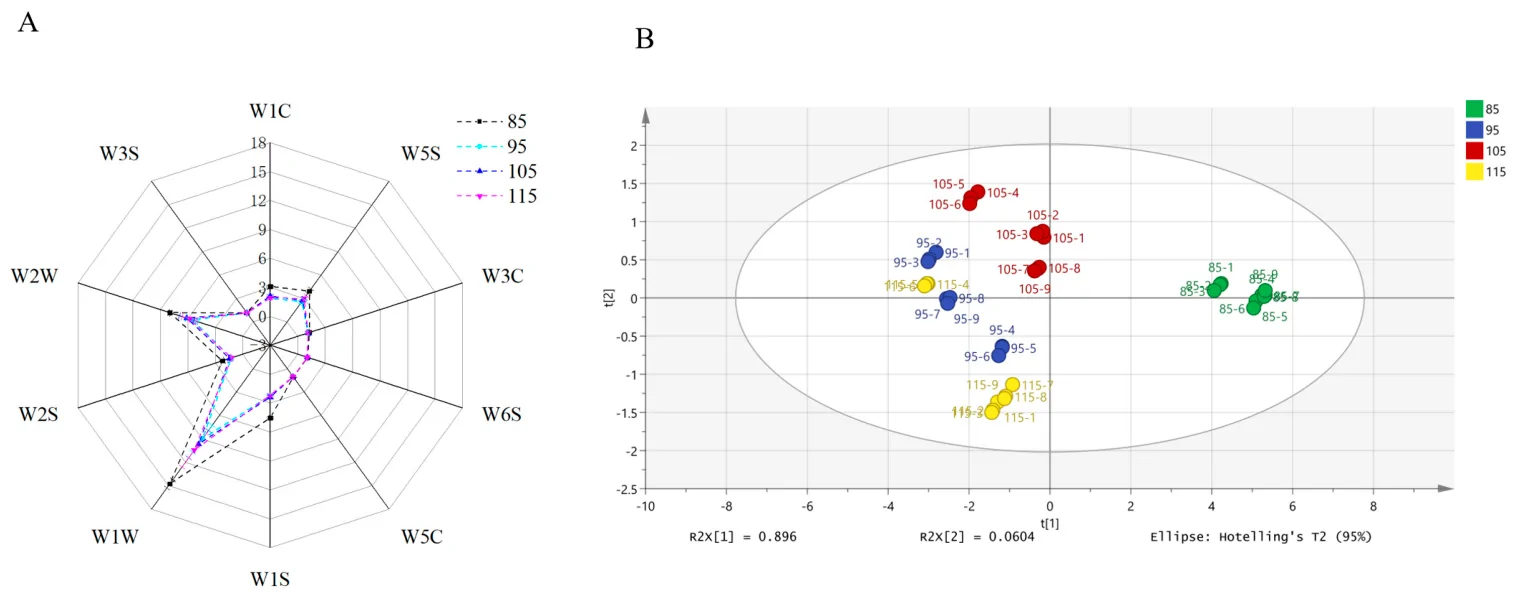
Electronic nose (E-nose) analysis of luncheon meat after heat treatment at different temperatures. (**A**) Radar plot of the response values of the 10 E-nose sensors (sensor assignments are listed in Table 2); a larger value along each axis indicates a stronger sensor response. (**B**) Principal component analysis (PCA) score plot of the E-nose sensor responses. Three stable data points were extracted from the steady region of the characteristic curve obtained from each independent replicate, and three independent replicates were prepared for each treatment group.

As shown in Figure 2A, the volatile odors of luncheon meat treated at different temperatures were generally similar, with only slight differences in the intensity and proportions of various volatiles. During the steady-state phase, the sensors showing the strongest responses were W1W and W2W. According to Table 2, they were sensitive to sulfides and aromatic components/organic sulfides, respectively, indicating that sulfur-containing volatile substances significantly contributed to the overall odor of the luncheon meat. Principal component analysis (PCA) was applied to analyze the response signals of luncheon meat samples treated at different temperatures (Figure 2B). The combined contribution of principal components reached 95.6% (PC1: 89.6%, PC2: 6.0%), which covered most of the original information of the samples. The clear separation of the 85 and 115 °C groups along PC1 suggests that the E-nose can effectively discriminate between products processed at different temperatures.

### 3.7. MDA Content

Table 3 presents the effects of heat treatment on the lipid oxidation stability of luncheon meat, as indicated by the malondialdehyde (MDA) content. The MDA content increased significantly with increasing temperature, rising from 0.34 mg/kg at 85 °C to 0.69 mg/kg at 115 °C. Elevated processing temperatures promote the cleavage of polyunsaturated fatty acid hydroperoxides, thereby facilitating the formation of secondary oxidation products such as MDA [22,44]. Previous studies have demonstrated that the increase in TBARS values is consistent with the changes in the total aldehyde content [45]. As shown in Table 7, the content of aldehyde-derived volatile compounds in the samples exhibited an increasing trend with increasing cooking temperature, further indicating that the generation of aldehydic volatiles is closely associated with lipid oxidation.

### 3.8. VOCs

**Figure 3:**
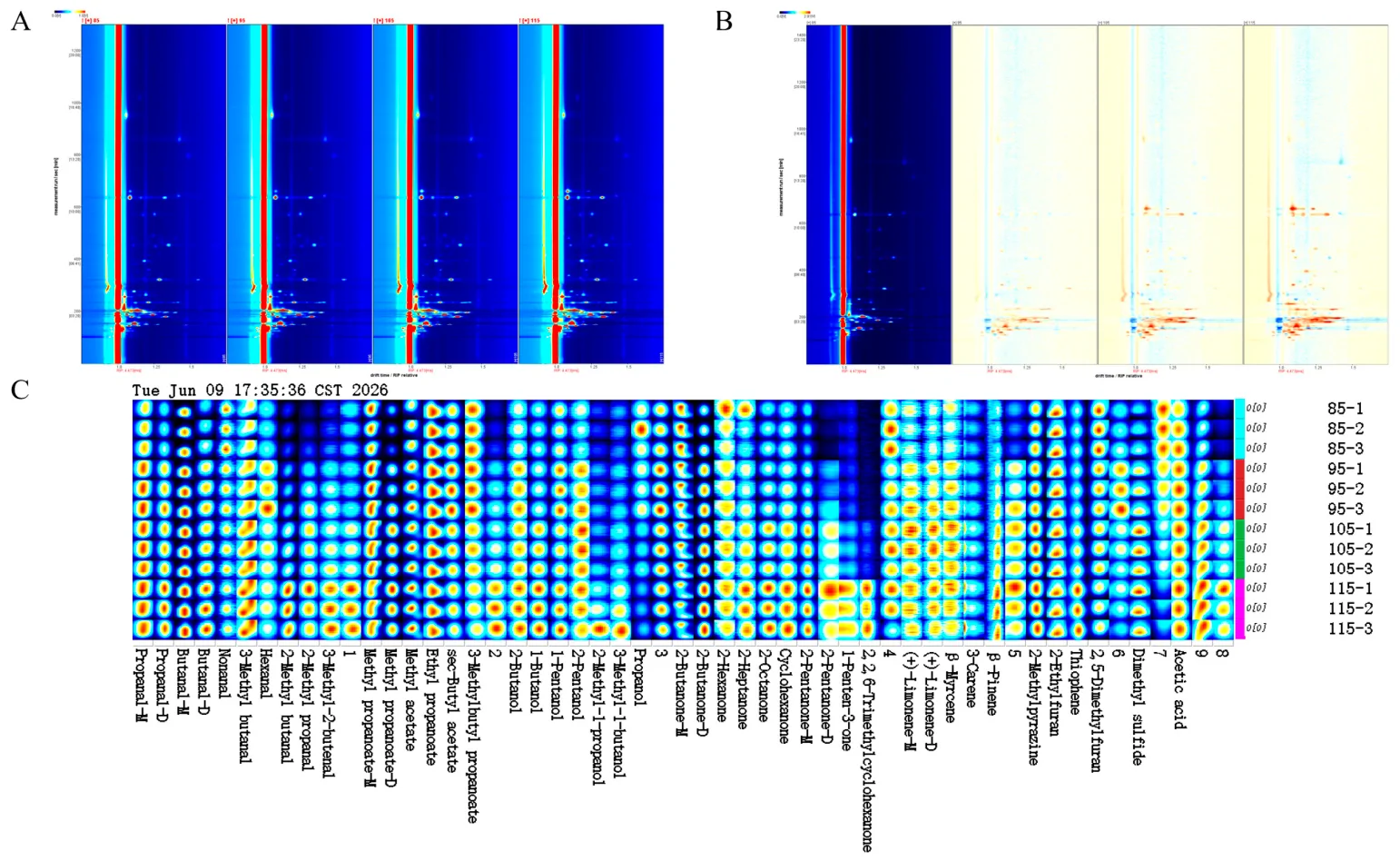
GC-IMS spectra of luncheon meat after heat treatment at different temperatures. (**A**) Two-dimensional spectrum of the sample. (**B**) Volatile fingerprint difference spectrum, using the 85 °C sample as the reference. (**C**) Fingerprint spectrum of the sample. Note: Different colors represent the relative abundance of volatile compounds, and the brighter the color, the higher the content. The gradient from red to blue represents that the concentration of this compound is higher (in red) or lower (in blue) compared to the reference sample. X-axis: drift time/RIP relative (range: 1.0–1.8). Y-axis: measurement run time in seconds (range: 0–1200 s for (**A**); 0–1400 s for (**B**)).

**Figure 4:**
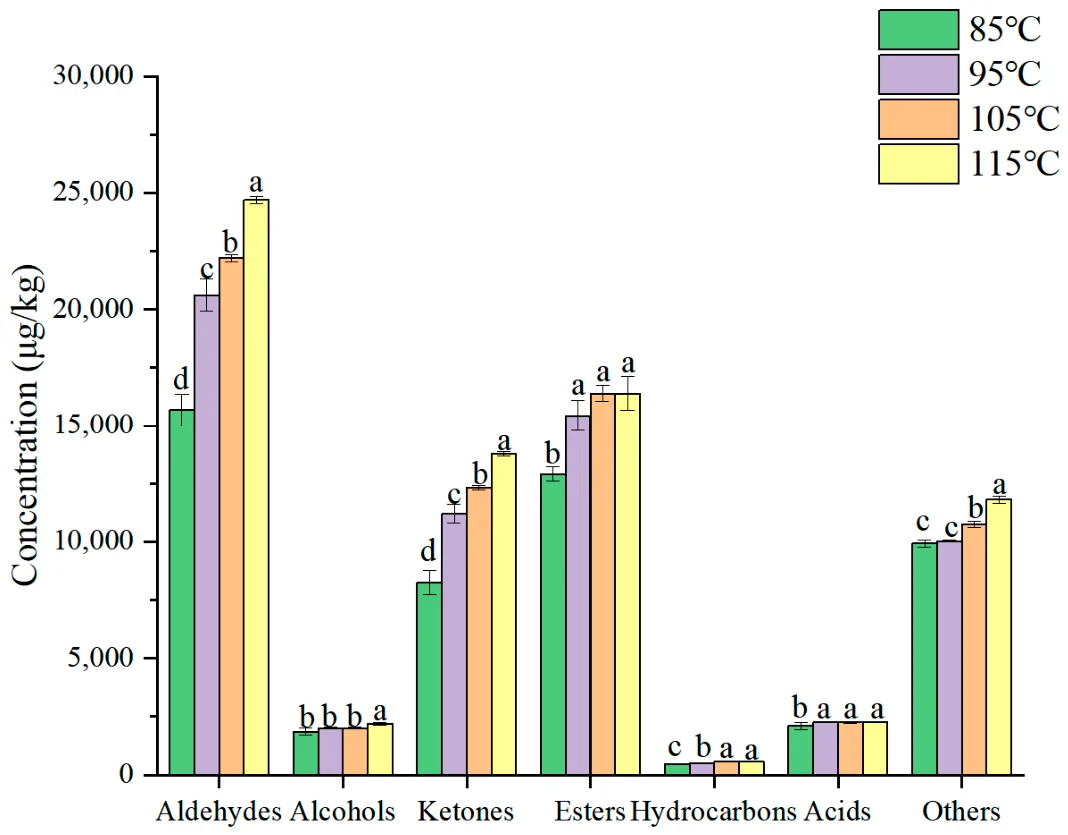
Total contents of volatile organic compounds (VOCs) of different chemical classes (e.g., aldehydes, ketones, alcohols, esters, and terpenes) in luncheon meat after heat treatment at 85, 95, 105, and 115 °C. Contents are given in μg/kg, and data are expressed as mean ± standard deviation (SD) of three independent replicates (n = 3). Different lowercase superscript letters indicate significant differences (*p* < 0.05), whereas values sharing the same letter do not differ significantly.

GC-IMS offers advantages such as extremely high sensitivity, spectral visualization, and simple sample preparation. The two-dimensional spectra (Figure 3A) retain the chromatographic retention time, ion drift time, and peak response intensity, thereby enabling rapid determination of inter-group differences [8]. Using the 85 °C spectrum as a reference, the difference spectrum (Figure 3B) was obtained. The gradient from red to blue represents that the concentration of this compound is higher (in red) or lower (in blue) compared to the reference sample. As observed from Figure 3B, the contents of VOCs increased with the rise in temperature. To clearly illustrate the differences in VOCs between each group, corresponding fingerprint spectra (Figure 3C) were generated. In total, 38 volatile compounds were successfully identified (monomers and dimers were counted as one substance), while 9 substances remained unidentified. All identified compounds were classified into seven categories, including 8 aldehydes, 7 alcohols, 8 ketones, 5 esters, 4 hydrocarbons, 1 acid and 5 other compounds. The types of substances were consistent with the research results of Zhu et al. [8] on the flavor of pork emulsion sausage. As shown in Figure 4, aldehydes and esters dominated the VOC profiles of luncheon meat, followed by ketones, whereas alcohols and alkenes exhibited the lowest relative contents. The contents of all categories of volatile compounds at 105 °C and 115 °C were significantly higher than those at 85 °C, demonstrating that thermal processing regulates flavor formation. The specific changes in VOCs content are shown in Table 7.

Aldehydes and ketones are abundant volatile compounds in the samples, primarily derived from the oxidation of unsaturated fatty acids, amino acid degradation, and Strecker reactions [46]. Among ketones, 2-octanone increased from 857.49 to 1521.57 μg/kg, contributing fatty and mushroom-like notes. The total content of aldehydes showed a continuous upward trend with the increase in heat treatment temperature, rising from 15,677.98 μg/kg at 85 °C to 24,687.25 μg/kg at 115 °C. The contents of aliphatic aldehydes (propanal, butanal, hexanal) increased markedly, indicating accelerated oxidative degradation of lipids at elevated temperatures. Butanal, the most abundant aldehyde, increased from 10,547.63 μg/kg to 15,514.09 μg/kg. Previous studies have confirmed that linoleic acid is the predominant polyunsaturated fatty acid in pork, and hexanal is primarily generated from the oxidative degradation of linoleic acid [47]. Branched-chain aldehydes (2-methylpropanal, 2-methylbutanal) also increased significantly, as these are characteristic products of Strecker degradation, and higher temperatures promote the Maillard reaction between reducing sugars and amino acids [21]. 2-methylbutanal, a key Strecker degradation product, showed the most dramatic increase (488.51 to 2217.31 μg/kg), indicating that the Maillard reaction was strongly temperature-dependent. Alcohols are mainly produced via lipid oxidative degradation and generally have relatively high odor thresholds, resulting in weaker contributions to meat flavor [48]. The content of alcohol compounds increased with the increase in cooking temperature, indicating that thermal treatment induced lipid degradation. Nevertheless, the content of propanol presented a decreasing trend with rising temperature, likely due to the oxidation of alcohols to aldehydes under high-temperature conditions [21]. Esters are synthesized through esterification between free fatty acids and alcohols [44,49], and increasing temperature significantly accelerates this reaction. The increased alcohol content at higher temperatures provides additional substrates for esterification. Terpenes, mainly originating from spice volatilization and thermal cracking of lipids, contribute citrus and woody notes to the meat flavor. From an industrial standpoint, the significant increase in MDA content and the 57.5% increase in total aldehydes from 85 to 115 °C indicate that while higher temperatures enhance flavor development, they also promote lipid oxidation. Therefore, in the industrial processing, when using higher heat treatment temperatures, the introduction of antioxidants should be considered.

**Table 7:** Effects of heat treatment temperature (85, 95, 105, and 115 °C, 20 min) on volatile organic compounds (VOCs) and their odor activity values (OAVs) in luncheon meat.

Count	Compound	Formula	Odor Threshold (μg/kg)	Concentration (μg/kg)				OAV			
				85 °C	95 °C	105 °C	115 °C	85 °C	95 °C	105 °C	115 °C
1	2-methylpropanal	C_4_H_8_O	1.5	236.02 ^c^ ± 59.45	625.97 ^b^ ± 113.55	792.94 ^a^ ± 22.91	891.00 ^a^ ± 113.60	157.35	417.31	528.63	594.00
2	Propanal	C_3_H_6_O	15.1	2606.77 ^c^ ± 129.54	3579.97 ^b^ ± 121.19	3600.11 ^b^ ± 15.69	3948.24 ^a^ ± 45.07	172.63	237.08	238.42	261.47
3	Butanal	C_4_H_8_O	9	10,547.63 ^d^ ± 423.49	13,537.91 ^c^ ± 383.59	14,329.36 ^b^ ± 153.86	15,514.09 ^a^ ± 74.79	1171.96	1504.21	1592.15	1723.79
4	3-methylbutanal	C_5_H_10_O	1.1	1096.46 ^c^ ± 25.32	1202.90 ^b^ ± 9.46	1304.82 ^a^ ± 8.36	1309.37 ^a^ ± 8.77	996.78	1093.55	1186.20	1190.34
5	2-methylbutanal	C_5_H_10_O	1	488.51 ^d^ ± 27.24	854.27 ^c^ ± 74.03	1380.24 ^b^ ± 29.08	2217.31 ^a^ ± 95.3	488.51	854.27	1380.24	2217.31
6	Hexanal	C_6_H_12_O	4.5	150.45 ^c^ ± 2.92	236.16 ^a^ ± 14.04	194.75 ^b^ ± 14.12	181.32 ^b^ ± 17.72	33.43	52.48	43.28	40.29
7	3-methyl-2-butenal	C_5_H_8_O	-	58.64 ^c^ ± 9.77	104.33 ^b^ ± 2.89	118.40 ^b^ ± 8.53	160.41 ^a^ ± 10.67	-	-	-	-
8	Nonanal	C_9_H_18_O	1	493.41 ^a^ ± 32.31	478.72 ^a^ ± 22.49	480.85 ^a^ ± 8.32	465.51 ^a^ ± 31.78	493.41	478.72	480.85	465.51
9	2-butanol	C_4_H_10_O	3300	73.81 ^b^ ± 2.10	90.86 ^a^ ± 0.65	91.27 ^a^ ± 4.66	97.60 ^a^ ± 5.43	0.02	0.03	0.03	0.03
10	Propanol	C_3_H_8_O	8505.6	620.27 ^a^ ± 130.09	545.02 ^a^ ± 22.92	398.24 ^b^ ± 34.25	307.09 ^b^ ± 6.55	0.07	0.06	0.05	0.04
11	2-methyl-1-propanol	C_4_H_10_O	6505.2	39.38 ^b^ ± 4.72	54.21 ^b^ ± 3.32	55.51 ^b^ ± 8.20	90.41 ^a^ ± 26.07	<0.01	<0.01	<0.01	0.01
12	2-pentanol	C_5_H_12_O	8100	66.24 ^b^ ± 8.21	74.42 ^ab^ ± 2.04	81.99 ^a^ ± 4.72	82.26 ^a^ ± 2.91	<0.01	<0.01	0.01	0.01
13	1-butanol	C_4_H_10_O	459.2	533.32 ^d^ ± 8.81	590.59 ^c^ ± 4.40	789.17 ^b^ ± 12.75	890.41 ^a^ ± 19.06	1.16	1.29	1.72	1.94
14	3-methyl-1-butanol	C_5_H_12_O	4	94.85 ^c^ ± 3.46	113.43 ^c^ ± 1.34	136.42 ^b^ ± 4.76	181.87 ^a^ ± 22.42	23.71	28.36	34.11	45.47
15	1-pentanol	C_5_H_12_O	150.2	437.2 ^c^ ± 13.10	540.55 ^a^ ± 12.62	467.77 ^b^ ± 15.93	547.24 ^a^ ± 9.40	2.91	3.60	3.11	3.64
16	2-butanone	C_4_H_8_O	35,400.2	6210.63 ^c^ ± 511.90	8735.77 ^b^ ± 330.18	9157.11 ^b^ ± 193.62	9999.86 ^a^ ± 121.56	0.18	0.25	0.26	0.28
17	2-pentanone	C_5_H_10_O	1380	458.01 ^d^ ± 41.43	677.15 ^c^ ± 51.56	891.47 ^b^ ± 31.65	987.30 ^a^ ± 54.46	0.33	0.49	0.65	0.72
18	1-penten-3-one	C_5_H_8_O	23	84.29 ^c^ ± 2.67	89.04 ^c^ ± 4.43	119.79 ^b^ ± 4.58	225.57 ^a^ ± 13.98	3.66	3.87	5.21	9.81
19	2-hexanone	C_6_H_12_O	560	147.24 ^a^ ± 18.26	155.18 ^a^ ± 8.02	161.24 ^a^ ± 5.36	167.24 ^a^ ± 9.54	0.26	0.28	0.29	0.30
20	2-heptanone	C_7_H_14_O	140	117.09 ^a^ ± 29.36	113.96 ^a^ ± 6.34	128.30 ^a^ ± 3.07	141.11 ^a^ ± 4.92	0.84	0.81	0.92	1.01
21	Cyclohexanone	C_6_H_10_O	280	362.23 ^d^ ± 31.97	425.11 ^c^ ± 20.50	521.21 ^b^ ± 12.52	591.38 ^a^ ± 13.52	1.29	1.52	1.86	2.11
22	2-octanone	C_8_H_16_O	50.2	857.49 ^d^ ± 56.59	983.63 ^c^ ± 31.48	1286.61 ^b^ ± 58.47	1521.57 ^a^ ± 35.75	17.08	19.59	25.63	30.31
23	2,2,6-trimethylcyclohexanone	C_9_H_16_O	100	16.82 ^c^ ± 2.29	23.13 ^c^ ± 1.47	42.64 ^b^ ± 3.46	132.16 ^a^ ± 7.77	0.17	0.23	0.43	1.32
24	Methyl acetate	C_3_H_6_O_2_	1500	535.59 ^c^ ± 32.69	697.31 ^b^ ± 22.29	707.95 ^b^ ± 8.78	760.66 ^a^ ± 9.87	0.36	0.46	0.47	0.51
25	Methyl propanoate	C_4_H_8_O_2_	4600	2877.52 ^c^ ± 377.84	4909.54 ^b^ ± 620.45	6231.92 ^a^ ± 269.39	6672.86 ^a^ ± 572.48	0.63	1.07	1.35	1.45
26	Ethyl propanoate	C_5_H_10_O_2_	10	7952.31 ^b^ ± 35.75	8068.76 ^a^ ± 32.16	7886.52 ^b^ ± 74.88	7468.29 ^c^ ± 72.94	795.23	806.88	788.65	746.83
27	sec-Butyl acetate	C_6_H_12_O_2_	6	1028.06 ^c^ ± 20.57	1242.45 ^a^ ± 31.48	1121.03 ^b^ ± 22.56	1094.90 ^b^ ± 52.88	171.34	207.08	186.84	182.48
28	3-methylbutyl propanoate	C_8_H_16_O_2_	8.6	516.49 ^a^ ± 2.90	525.43 ^a^ ± 11.10	454.48 ^b^ ± 9.66	397.02 ^c^ ± 32.25	60.06	61.10	52.85	46.17
29	β-pinene	C_10_H_16_	140	176.88 ^d^ ± 8.59	197.67 ^c^ ± 5.28	209.85 ^b^ ± 3.75	226.53 ^a^ ± 3.20	1.26	1.41	1.50	1.62
30	3-carene	C_10_H_16_	-	140.12 ^c^ ± 13.99	162.08 ^b^ ± 0.64	196.02 ^a^ ± 12.23	197.68 ^a^ ± 5.34	-	-	-	-
31	β-myrcene	C_10_H_16_	1.20	36.68 ^c^ ± 1.25	40.46 ^bc^ ± 2.53	45.61 ^a^ ± 2.99	45.17 ^ab^ ± 3.01	30.57	33.72	38.01	37.64
32	(+)-limonene	C_10_H_16_	10	103.14 ^b^ ± 4.71	110.48 ^ab^ ± 2.82	114.58 ^a^ ± 4.39	110.11 ^ab^ ± 5.73	10.31	11.05	11.46	11.01
33	Acetic acid	C_2_H_4_O_2_	22,000	2100.07 ^b^ ± 139.50	2255.53 ^a^ ± 18.22	2258.83 ^a^ ± 33.32	2266.22 ^a^ ± 27.69	0.10	0.10	0.10	0.10
34	Dimethyl sulfide	C_2_H_6_S	0.30	164.43 ^c^ ± 15.09	230.60 ^b^ ± 4.75	276.25 ^a^ ± 14.46	259.45 ^a^ ± 12.68	548.10	768.67	920.83	864.83
35	2,5-dimethylfuran	C_6_H_8_O	-	767.31 ^a^ ± 7.00	662.13 ^b^ ± 9.76	626.27 ^c^ ± 3.84	563.77 ^d^ ± 10.34	-	-	-	-
36	2-ethylfuran	C_6_H_8_O	-	3406.87 ^a^ ± 23.69	3262.02 ^b^ ± 45.75	3216.67 ^b^ ± 40.15	3206.84 ^b^ ± 80.55	-	-	-	-
37	Thiophene	C_4_H_4_S	84	2102.87 ^c^ ± 19.58	2202.10 ^c^ ± 43.39	2548.11 ^b^ ± 26.32	3367.79 ^a^ ± 91.78	25.03	26.22	30.33	40.09
38	2-methylpyrazine	C_5_H_6_N_2_	30,000	3486.92 ^d^ ± 99.13	3682.00 ^c^ ± 62.05	4089.80 ^b^ ± 58.44	4408.18 ^a^ ± 64.85	0.12	0.12	0.14	0.15

Note: Values are expressed as mean ± standard deviation (SD) of three independent replicates (n = 3). OAV, odor activity value, calculated as the ratio of the concentration of a volatile compound to its odor threshold. The odor thresholds were obtained from the book Compilations of Odor Threshold Values in Air, Water and Other Media (second enlarged and revised edition), and the threshold selected for each compound corresponds to the value measured in water. Different lowercase superscript letters within the same row indicate significant differences (*p* < 0.05), whereas values sharing the same letter do not differ significantly.

### 3.9. OAV Analysis

**Figure 5:**
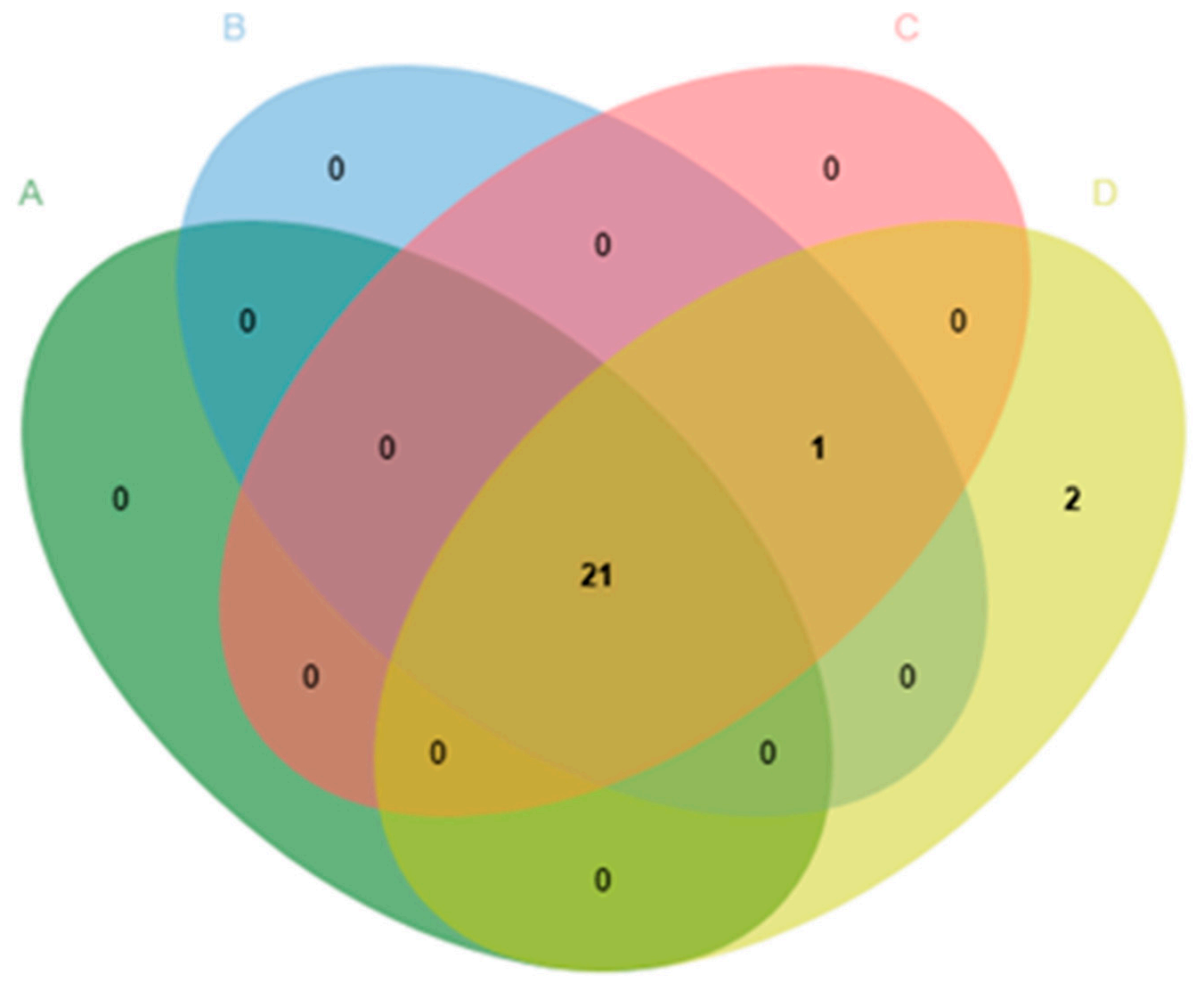
Venn diagram showing the distribution of key flavor-active volatile compounds (OAV > 1) in luncheon meat across four heat treatment temperatures. A = 85 °C, B = 95 °C, C = 105 °C, D = 115 °C. Each circle represents the set of compounds with OAV > 1 at the corresponding temperature. Overlapping regions indicate compounds shared among treatment groups, while non-overlapping regions represent temperature-specific flavor-active compounds.

The flavor characteristics of the product are not only determined by the content of volatile compounds, but also closely affected by their flavor thresholds. Generally, volatile compounds with OAV > 1 are regarded as key contributors to sample flavor, and a higher OAV value indicates a more prominent dominant effect on the overall flavor of products [50]. In this study, there were a total of 24 flavor activity substances with OAV > 1 in the samples treated at different temperatures, among which 21 substances were common in all four treatment groups (Figure 5). As can be seen from the table, aldehydes were the predominant flavor-active compounds, which was consistent with the finding reported by Li et al. [33]. 2-Methylbutanal, a Strecker degradation product of isoleucine, contributes malty and almond notes and was therefore a major driver of the intensified roasted aroma at higher temperatures. Butanal, derived primarily from lipid oxidation, imparts fatty and green notes. Ketones, represented mainly by 2-octanone, also increased with temperature and contributed milk, cheese, and mushroom notes. Dimethyl sulfide, despite its relatively low concentration, exhibited a high OAV values (548.10–920.83) due to its extremely low odor threshold (0.3 μg/kg), indicating its substantial contribution to the overall aroma profile. Alcohols, by contrast, generally possessed high odor thresholds and consequently made limited contributions to the aroma despite detectable concentration changes.

**Figure 6:**
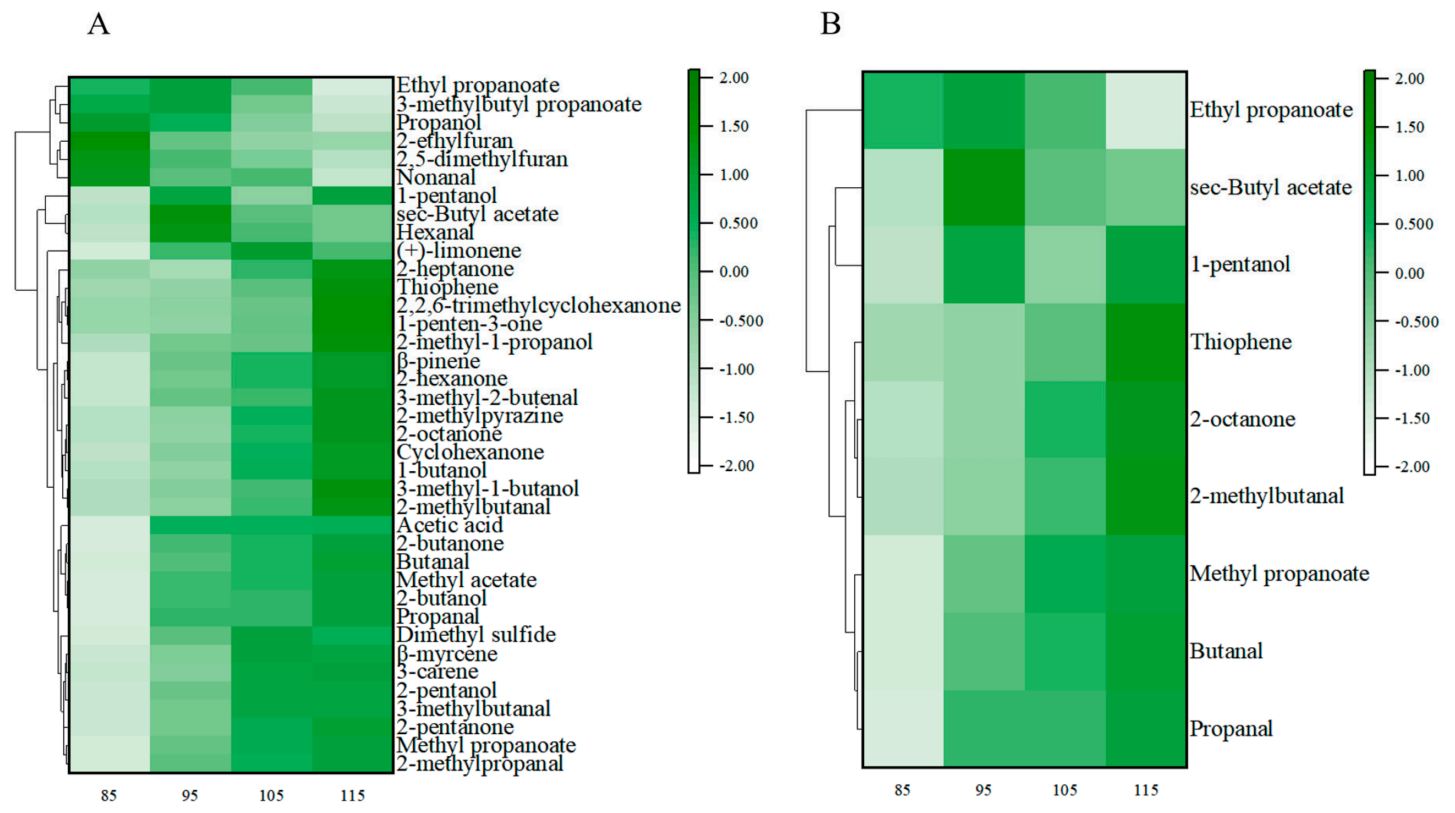
Clustering heatmaps of volatile compounds in luncheon meat samples after heat treatment at different temperatures. (**A**) All detected volatile compounds; (**B**) volatile compounds satisfying both OAV > 1 and variable importance in projection (VIP) > 1. The color gradient from white to green represents an increase in relative signal intensity. OAV, odor activity value; VIP, variable importance in projection.

To visualize the effect of temperature on the VOCs, the VOCs obtained by GC-IMS were visualized to generate two heatmaps (Figure 6). The color gradient from white to green represented an increase in relative intensity. The signal intensities of aldehydes (e.g., 2-methylbutanal, butanal) and ketones (e.g., 2-octanone) progressively increased from 85 to 115 °C, consistent with the OAV-based observations. The results showed that the VOCs in luncheon meat changed significantly after thermal treatment at different temperatures, demonstrating that temperature served as a critical variable affecting the formation and release of flavor substances.

### 3.10. OPLS-DA

**Figure 7:**
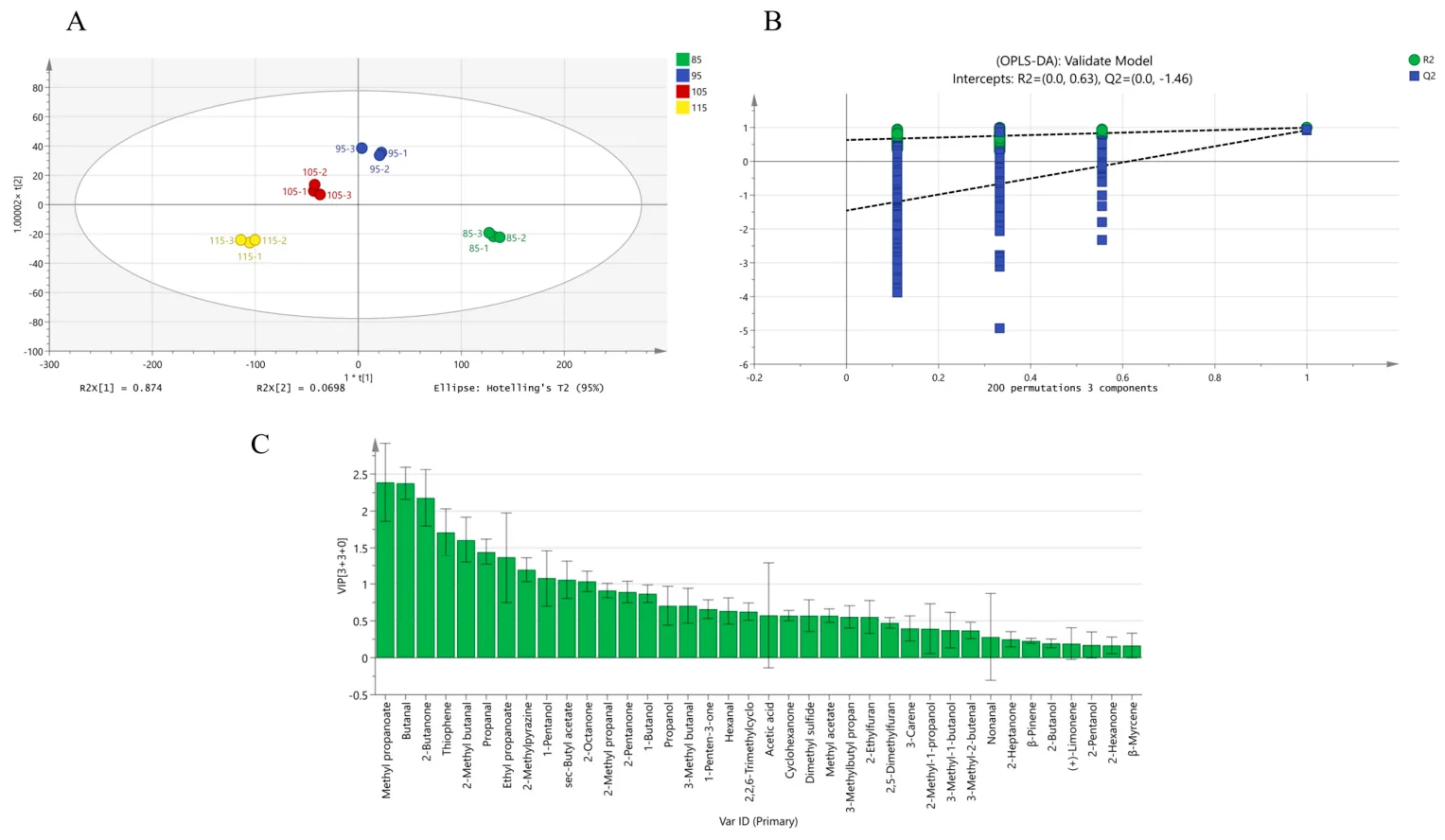
Orthogonal partial least squares discriminant analysis (OPLS-DA) of the volatile compound (GC-IMS) data of luncheon meat after heat treatment at different temperatures. (**A**) OPLS-DA score plot; (**B**) cross-validation of the model by a 200-permutation test; (**C**) variable importance in projection (VIP) score plot, where compounds with VIP > 1 are considered the main contributors to discrimination among treatment groups.

OPLS-DA of the full GC-IMS dataset produced a well-fitted model (R_2_ X = 0.995, R_2_ Y = 0.992, Q_2_ = 0.927; Figure 7A). The model was constructed from the GC-IMS data of three independent experimental replicates per treatment (12 samples in total). The 200 permutation tests showed that the y-intercept of the Q2 regression line was −1.46 (less than 0), confirming that the model did not overfit (Figure 7B). The key volatile compounds were identified by integrating two complementary criteria: (i) OAV > 1, indicating a potential contribution to the flavor profile (Table 7), and (ii) VIP > 1 in the OPLS-DA model (Figure 7C), indicating a significant contribution to the discrimination among treatment groups. Among the 11 compounds with VIP > 1, 9 also satisfied OAV > 1, thus being recognized as the key flavor-active compounds of luncheon meat (Figure 6B). Specifically, these include: methyl propanoate (fruit, rum), butanal (fruity, green leaf), thiophene (garlic, sulfur), 2-methyl butanal (almond, cocoa), propanal (pungent, green grassy), ethyl propanoate (fruity, rum), 1-pentanol (balsamic), sec-butyl acetate (fruity), and 2-octanone (cheese, mushroom). These compounds contribute diverse aroma notes, and their relative proportions varied systematically with temperature, driving the discrimination among treatment groups in the OPLS-DA model. The identification of 9 key flavor-active compounds provides specific targets for industrial flavor quality monitoring and process optimization.

## 4. Conclusions

This study found that heat treatment temperature significantly influences the quality and flavor of luncheon meat. The increased weight loss rate and decreased immobilized water content indicated that thermal treatment reduced the water-holding capacity of proteins and other components. Texture analysis revealed that higher temperatures reduced the hardness and chewiness of the luncheon meat. In addition, the color of luncheon meat darkened with a significant increase in redness value under high-temperature conditions, which was mainly attributed to myoglobin oxidation and the intensification of the Maillard reaction. With the increase in temperature, the content of MDA significantly increased, indicating the enhancement of lipid oxidation. The results of GC-IMS and E-nose analysis illustrated that the contents of characteristic volatile compounds, including aldehydes, ketones and esters, were markedly enhanced with the increase in temperature, which indicated that high temperature significantly promoted lipid oxidation. OAV analysis and OPLS-DA modeling converged on 9 key flavor-active compounds—butanal, 2-methyl butanal, propanal, ethyl propanoate, methyl propanoate, thiophene, 1-pentanol, sec-butyl acetate, and 2-octanone—as primary discriminators of temperature-dependent flavor variation. It should be noted that no sensory evaluation was performed in this study; therefore, the flavor differences established through instrumental analyses (GC-IMS, E-nose, and OAV calculation) have not been validated against human perception, and no conclusions can be drawn regarding the sensory acceptability and overall consumer preference of luncheon meat subjected to different sterilization temperatures. Further research should incorporate quantitative descriptive sensory analysis and consumer acceptance testing, microbial safety assessment, systematically investigate the changes in fat and protein structures, and optimize the time and temperature parameters to comprehensively validate and supplement the theoretical basis for regulating the overall quality of luncheon meat through thermal treatment.

## Figures and Tables

**Table 1:** Basic formula for luncheon meat.

Ingredient	Percentage (%)	Amount (g)
Pork foreleg meat	71.19	740
Pork fat	10.58	110
Strains	0.01	0.08
Ice water	9.62	100
Corn starch	3.37	35
Sugar	1.92	20
Salt	1.25	13
Seasoning	0.77	8
Collagen protein seasoning	0.48	5
Transglutaminase	0.41	4.25
Compound phosphates	0.29	3
White pepper	0.08	0.8
Nisin	0.03	0.3

Note: The strain is *Staphylococcus carnosus* and *Mammaliicoccus vitulinus*. The seasoning is a commercial blend whose main ingredients include trehalose, sodium D-isoascorbate, beet juice powder, salt, and maltodextrin as a carrier. The collagen protein seasoning is a commercial blend whose main ingredients include collagen, maltodextrin, soup powder, and salt. The complex phosphate salts include sodium pyrophosphate, sodium tripolyphosphate and sodium hexametaphosphate, with a ratio of 1:1:1.

**Table 2:** Performance specifications of the E-nose sensors.

Sensor Number	Sensors Name	Sensitive Substance
1	W1C	Aromatic compounds
2	W5S	Nitrogen oxides
3	W3C	Ammonia, aromatic molecule
4	W6S	Hydrides
5	W5C	Olefin, aromatic components, and polar molecules
6	W1S	Alkanes
7	W1W	Sulfides
8	W2S	Alcohols and some aromatic compounds
9	W2W	Aromatic components and organic sulfides
10	W3S	Aliphatic series

**Table 3:** Effects of heat treatment temperatures (85, 95, 105 and 115 °C, for 20 min) on the weight loss rate (%), color parameters (L*, a*, b*) and malondialdehyde (MDA) content of luncheon meat.

Temperature (°C)	Weight Loss (%)	L*	a*	b*	MDA (mg/kg)
85	2.63 ^c^ ± 0.15	59.40 ^a^ ± 0.51	5.69 ^c^ ± 0.18	10.59 ^c^ ± 0.24	0.34 ^c^ ± 0.07
95	2.66 ^c^ ± 0.17	58.37 ^ab^ ± 0.88	5.77 ^c^ ± 0.05	10.23 ^c^ ± 0.26	0.44 ^bc^ ± 0.07
105	3.01 ^b^ ± 0.03	59.33 ^ab^ ± 0.56	6.25 ^b^ ± 0.02	11.64 ^b^ ± 0.33	0.49 ^b^ ± 0.09
115	3.26 ^a^ ± 0.02	58.05 ^b^ ± 0.66	6.77 ^a^ ± 0.17	12.28 ^a^ ± 0.19	0.69 ^a^ ± 0.02

Note: Values are expressed as mean ± standard deviation (SD) of three independent replicates (n = 3). L*, a*, and b* are the CIELAB color parameters representing lightness, redness/greenness, and yellowness/blueness, respectively. MDA, malondialdehyde. Different lowercase superscript letters within the same column indicate significant differences (*p* < 0.05), whereas values sharing the same letter do not differ significantly.

**Table 4:** Effects of heat treatment temperatures (85, 95, 105, and 115 °C, for 20 min) on the texture analysis (TPA) parameters of luncheon meat.

Temperature (°C)	Hardness	Cohesiveness	Springiness	Gumminess	Chewiness
85	1615.71 ^a^ ± 79.74	0.73 ^b^ ± 0.01	3.30 ^b^ ± 0.09	1182.00 ^a^ ± 53.15	3908.00 ^a^ ± 206.14
95	1527.00 ^b^ ± 115.47	0.75 ^a^ ± 0.01	3.44 ^b^ ± 0.20	1151.57 ^a^ ± 85.59	3958.71 ^a^ ± 351.46
105	1283.00 ^c^ ± 44.30	0.70 ^c^ ± 0.02	3.77 ^a^ ± 0.14	903.86 ^b^ ± 32.08	3404.43 ^b^ ± 63.44
115	1052.86 ^d^ ± 19.56	0.69 ^d^ ± 0.01	3.68 ^a^ ± 0.07	722.86 ^c^ ± 18.19	2660.14 ^c^ ± 82.70

Note: Values are expressed as mean ± standard deviation (SD) of ten replicates (n = 10). TPA, texture profile analysis. Different lowercase superscript letters within the same column indicate significant differences (*p* < 0.05), whereas values sharing the same letter do not differ significantly.

**Table 5:** Effects of heat treatment temperatures (85, 95, 105 and 115 °C for 20 min) on the water distribution of luncheon meat, as determined by low-field nuclear magnetic resonance (LF-NMR) relaxometry.

Temperature (°C)	A_21_ (%)	A_22_ (%)	A_23_ (%)
85	3.68 ^c^ ± 0.05	95.20 ^a^ ± 0.10	1.12 ^c^ ± 0.05
95	3.86 ^b^ ± 0.05	94.67 ^b^ ± 0.13	1.47 ^b^ ± 0.13
105	4.23 ^a^ ± 0.03	93.50 ^c^ ± 0.05	2.26 ^a^ ± 0.08
115	4.23 ^a^ ± 0.16	93.60 ^c^ ± 0.10	2.17 ^a^ ± 0.24

Note: Values are expressed as mean ± standard deviation (SD) of three independent replicates (n = 3). A_21_, A_22_, and A_23_ represent the proportions of bound water, immobilized water, and free water, respectively, obtained from the LF-NMR transverse relaxation (T2) analysis. Different lowercase superscript letters within the same column indicate significant differences (*p* < 0.05), whereas values sharing the same letter do not differ significantly.

**Table 6:** Effects of heat treatment temperatures (85, 95, 105, and 115 °C, for 20 min) on the amino acid composition of luncheon meat.

Amino Acid	Concentration (g/100 g)			
	85 °C	95 °C	105 °C	115 °C
Tyr	0.41 ^a^ ± 0.02	0.43 ^a^ ± 0.01	0.43 ^a^ ± 0.02	0.43 ^a^ ± 0.02
Ser	0.60 ^b^ ± 0.02	0.64 ^a^ ± 0.01	0.63 ^ab^ ± 0.01	0.62 ^ab^ ± 0.02
Glu	2.24 ^b^ ± 0.06	2.43 ^a^ ± 0.04	2.38 ^a^ ± 0.01	2.37 ^a^ ± 0.08
Gly	0.89 ^a^ ± 0.02	0.97 ^a^ ± 0.03	0.97 ^a^ ± 0.06	0.95 ^a^ ± 0.04
Ala	0.95 ^b^ ± 0.03	1.03 ^a^ ± 0.02	1.01 ^a^ ± 0.03	1.00 ^a^ ± 0.03
His	0.52 ^b^ ± 0.01	0.55 ^a^ ± 0.01	0.54 ^ab^ ± 0.02	0.54 ^ab^ ± 0.01
Pro	0.62 ^a^ ± 0.01	0.67 ^a^ ± 0.03	0.65 ^a^ ± 0.04	0.64 ^a^ ± 0.03
Arg	0.96 ^b^ ± 0.03	1.03 ^a^ ± 0.03	1.00 ^ab^ ± 0.03	1.00 ^ab^ ± 0.03
Asp	1.24 ^b^ ± 0.04	1.35 ^a^ ± 0.02	1.33 ^a^ ± 0.01	1.33 ^a^ ± 0.03
Val	0.69 ^c^ ± 0.02	0.76 ^a^ ± 0.01	0.73 ^b^ ± 0.01	0.74 ^ab^ ± 0.02
Thr	0.66 ^b^ ± 0.02	0.72 ^a^ ± 0.01	0.70 ^a^ ± 0.01	0.70 ^a^ ± 0.02
Phe	0.63 ^b^ ± 0.02	0.68 ^a^ ± 0.01	0.66 ^ab^ ± 0.01	0.66 ^ab^ ± 0.02
Ile	0.69 ^b^ ± 0.01	0.74 ^a^ ± 0.01	0.72 ^ab^ ± 0.02	0.74 ^a^ ± 0.03
Leu	1.14 ^b^ ± 0.03	1.24 ^a^ ± 0.03	1.19 ^ab^ ± 0.02	1.19 ^ab^ ± 0.04
Lys	1.36 ^b^ ± 0.04	1.47 ^a^ ± 0.03	1.43 ^ab^ ± 0.05	1.40 ^ab^ ± 0.05
TEAA	5.17	5.61	5.43	5.43
TAA	13.60	14.71	14.37	14.31

Values are expressed as mean ± standard deviation (SD) of three independent replicates (n = 3). TEAA, total essential amino acids; TAA, total amino acids; the TEAA and TAA rows present the sums of the corresponding amino acid contents. Abbreviations: Tyr, tyrosine; Ser, serine; Glu, glutamic acid; Gly, glycine; Ala, alanine; His, histidine; Pro, proline; Arg, arginine; Asp, aspartic acid; Val, valine; Thr, threonine; Phe, phenylalanine; Ile, isoleucine; Leu, leucine; Lys, lysine. Different lowercase superscript letters within the same row indicate significant differences (*p* < 0.05), whereas values sharing the same letter do not differ significantly.

## Data Availability

The original contributions presented in the study are included in the article and Supplementary Materials, further inquiries can be directed to the corresponding authors.

## Supplementary Materials

The following supporting information can be downloaded at: https://www.mdpi.com/article/10.3390/foods15173131/s1, File S1: GC-IMS Instrument Parameters.
