# Supplementary material for: Temperature-Driven Textural Evolution, Quality Changes, and Flavor Development in Luncheon Meat
Source: Foods. 2026 Sep 3;15(17):3131. doi: 10.3390/foods15173131 (PMC13564673; doi:10.3390/foods15173131)
Supplement: Supplementary file 1 [file foods-15-03131-s001.zip › foods-4474189-supplementary.pdf]

Supplementary Materials

File S1: GC-IMS Instrument Parameters：

The MXT-WAX chromatographic column (15m×0.53mm×1μm) was used for chromatographic separation at 60°C. The sample was incubated at 60°C for 15 min at a speed of 500 r/min, with an injection volume of 200 μL and a needle temperature of 105°C. The injection was carried out in the non-split mode using nitrogen as the carrier gas, with an injector temperature of 80°C. The carrier gas program was: 2.0 mL/min held for 2 min, ramped linearly to 10.0 mL/min over 8 min, then to 100.0 mL/min over 10 min, and held for 20 min. During this process, the ions were injected into the chromatographic tube at a flow rate of 75.0 mL/min at 45°C.
